# Bioconversion of High Concentrations of Hydrogen Sulfide to Elemental Sulfur in Airlift Bioreactor

**DOI:** 10.1155/2014/675673

**Published:** 2014-07-22

**Authors:** Mohamed Abdel-Monaem Zytoon, Abdulraheem Ahmad AlZahrani, Madbuli Hamed Noweir, Fadia Ahmed El-Marakby

**Affiliations:** ^1^Department of Industrial Engineering, King Abdulaziz University, P.O. Box 80204, Jeddah 21589, Saudi Arabia; ^2^Department of Occupational Health and Air Pollution, High Institute of Public Health, Alexandria University, 165 El Horreya Avenue, Alexandria, Egypt; ^3^Department of Chemical and Material Engineering, King Abdulaziz University, P.O. Box 80204, Jeddah 21589, Saudi Arabia; ^4^Center of Excellence for Environmental Studies, King Abdulaziz University, P.O. Box 80204, Jeddah 21589, Saudi Arabia

## Abstract

Several bioreactor systems are used for biological treatment of hydrogen sulfide. Among these, airlift bioreactors are promising for the bioconversion of hydrogen sulfide into elemental sulfur. The performance of airlift bioreactors is not adequately understood, particularly when directly fed with hydrogen sulfide gas. The objective of this paper is to investigate the performance of an airlift bioreactor fed with high concentrations of H_2_S with special emphasis on the effect of pH in combination with other factors such as H_2_S loading rate, oxygen availability, and sulfide accumulation. H_2_S inlet concentrations between 1,008 ppm and 31,215 ppm were applied and elimination capacities up to 113 g H_2_S m^−3^ h^−1^ were achieved in the airlift bioreactor under investigation at a pH range 6.5–8.5. Acidic pH values reduced the elimination capacity. Elemental sulfur recovery up to 95% was achieved under oxygen limited conditions (DO < 0.2 mg/L) and at higher pH values. The sulfur oxidizing bacteria in the bioreactor tolerated accumulated dissolved sulfide concentrations >500 mg/L at pH values 8.0–8.5, and near 100% removal efficiency was achieved. Overall, the resident microorganisms in the studied airlift bioreactor favored pH values in the alkaline range. The bioreactor performance in terms of elimination capacity and sulfur recovery was better at pH range 8–8.5.

## 1. Introduction

Hydrogen sulfide is emitted from many industrial activities. The toxicity, malodor, and corrosiveness of H_2_S necessitate its removal from waste gas streams. The classical physicochemical processes for H_2_S control have many drawbacks, such as large energy requirements, high capital and operating costs, and production of secondary wastes [1–7]. On the other hand, biological processes for the removal of H_2_S are more attractive because they are believed to be inexpensive and cause no environmental pollution [5].

Biofilters packed with compost and other natural media [8–12] and those with synthetic beds [2, 3, 5, 6, 13–19] have been studied. However, these types of biofilters are limited to air streams with low concentrations of H_2_S. In addition, they produce waste streams containing sulfate/sulfuric acid, which need further treatment.

Several trials have been conducted to convert H_2_S biologically into elemental sulfur (S^o^) that can be easily separated from the waste stream and further treated for marketing. In some of these, heterotrophic sulfide oxidizing bacteria (SOB) were used [20]. However, the cost of continuous organic carbon supply is one drawback of such process. Also, iron-based biological processes have been studied [21–24]. The process consisted of two reactors, which increased the capital and operating costs of the process.

Biotrickling filters with synthetic packing materials were studied for bioconversion of H_2_S to S^o^ using autotrophic SOB [1, 25–27]. However, the problem with these types of reactors is that the produced sulfur particles block pores of the packing material and increase back pressure in the bioreactor. Therefore, they may not be suitable for treatment of gas streams with high H_2_S concentrations or loads where large amount of elemental sulfur is expected to be produced, such as in case of air streams with high H_2_S concentrations (up to several hundred or few thousands ppm) and energy-rich gases such as biogas from anaerobic digesters or landfills which may contain H_2_S concentrations up to several thousand ppm.

Suspended-growth bioreactors have no packing materials. When seeded with autotrophic SOB, they can overcome the aforementioned drawbacks. Study of the application of these bioreactors for biological oxidation of sulfide to elemental sulfur has been reported [28–32]. In these studies the inlet streams were sulfide-containing solutions rather than H_2_S gas.

The objective of the current study was to study biological treatment of high concentrations of H_2_S in an airlift bioreactor where direct injection of H_2_S gas into the bioreactor is applied, with special emphasis on the effect of pH in combination with other factors such as sulfide loading rate, oxygen availability, and sulfide accumulation.

## 2. Materials and Methods

### 2.1. Experimental Set-Up

**Figure 1:**
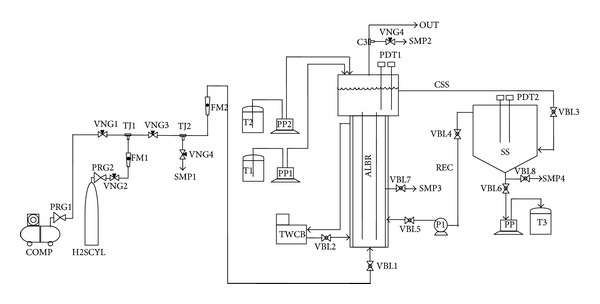
Schematic of the bioreactor system. ALBR: air-lift bioreactor; COMP: air compressor; CSS: cell and sulfur suspension; FM: flow meter; H_2_SCYL: H_2_S cylinder; OUT: outlet air to hood; P: circulation pump; PDT: pH/DO/Temp sensors; PP: peristaltic pump; PRG: pressure reducer and pressure gauge; REC: recycled cell suspension; SMP: gas/liquid sampling; SS: sulfur settler; T: tanks (1: nutrient; 2: HCl; and 3: sulfur sludge); TJ: tee joint; TWCB: thermostated water circulation bath; VNG: gas needle valve; and VBL: liquid ball valve.

The experimental set-up shown in Figure 1 consisted of three main sections: the H_2_S-air preparation section, the airlift bioreactor, and the sulfur settler. The airlift bioreactor consisted of two concentric 140 cm long acrylic tubes: the draft tube and the downcomer tube. The inside diameters of the two tubes were 6 cm and 15 cm, respectively. The working volume was 24.75 liters. The phase separator was 30 cm long with 30 cm inside diameter and filled up to 50% of its height. The bioreactor was jacketed with a 20 cm inside diameter acrylic tube for temperature control inside the bioreactor. Several ports for inlet and outlet gas streams, nutrient supply, pH adjustment solutions, cell suspension circulation between the bioreactor and the settler, and pH/DO/temperature sensors existed.

The sulfur settler was constructed from an acrylic tube with 40 cm height and 40 cm inside diameter, fitted to a conical bottom with 20 cm height. The bioreactor solution was continuously withdrawn to the settler for separation of the formed sulfur and the supernatant from the settler was recycled to the bioreactor. The settled sulfur slurry was withdrawn from the bottom of the settler cone for further treatment.

Air was driven to the bioreactor by a compressor. Before entering the bioreactor bottom, air was mixed with a stream of H_2_S coming from a cylinder at a controlled flow rate to bring about a calculated H_2_S concentration. Gas flow meters (Cole-Parmer EW-3227-08/28) were used to control air and H_2_S flow rates and, consequently, H_2_S concentrations.

### 2.2. Microbial Culture and Operation of the Bioreactor

The bioreactor was inoculated with 0.5 kg of activated sludge from Bani Malik Sewage Treatment Plant. A mixed culture of SOB was enriched using a thiosulfate nutrient solution for increasing biomass yield. The composition of the medium (in g/L) inside the bioreactor was as follows [4]: Na_2_HPO_4_ *·*7H_2_O: 2.27; KH_2_PO_4_: 1.8; MgCl_2_ *·*7H_2_O: 0.1; (NH_4_)_2_SO_4_: 1.98; MnCl_2_ *·*H_2_O: 0.023; CaCl_2_: 0.03; FeCl_3_ *·*6H_2_O: 0.033; Na_2_CO_3_: 1.0; and Na_2_S_2_O_3_ *·*5H_2_O: 15.69. Air was continuously supplied at a flow rate of 1.0 L/min without circulation of the bioreactor solution for 3 days, after which circulation of the resulting suspension was initiated between the airlift bioreactor and the settler with continuous addition of the thiosulfate mineral solution (5 mL/min) and withdrawal of the settled solids. Additional thiosulfate was added to the bioreactor on daily basis to insure sufficient supply for the developed SOB. When thiosulfate consumption rate by the developed SOB reached a maximum value, loading of H_2_S gas to the bioreactor started and the nutrient medium without thiosulfate was supplied.

During a period of 176 days of operation, the airlift bioreactor was fed with H_2_S as the sole sulfide source in predetermined concentrations (from 1,008 ppm to 31,215 ppm) in a continuous air stream of 1.0 liter/min. The inlet concentration of H_2_S was increased gradually to increase the sulfide loading rate (from 4.2 up to 132.4 g H_2_S m^−3^ h^−1^). The increase of H_2_S inlet concentration was on the expense of oxygen concentration, resulting in a decrease in dissolved oxygen. During a period of almost stable load and dissolved oxygen, the value of pH was changed. The pH value was controlled by adding HCl or Na_2_CO_3_. The temperature of the bioreactor was controlled at 30°C most of the time.

### 2.3. Abiotic Experiment

The abiotic experiments were conducted by adding sterilized activated sludge to the nutrient solution in the bioreactor and H_2_S-air mixture (about 1000 ppm) was introduced to the bioreactor for three days. During the first few hours of the first day the removal efficiency was high and then sharply decreased to a maximum of 3% during the remaining period. Analysis of the bioreactor solution revealed accumulation of the sulfide in the bioreactor solution without formation of elemental sulfur. Only very slight increase in sulfate (1.3%) and thiosulfate (0.82%) over their original concentrations was observed.

### 2.4. Chemical Analysis

Sulfur species (sulfate, thiosulfate, sulfide, and elemental sulfur) were measured in the outlet liquid solution on a daily basis. Barium sulfate turbidimetric method [33] was used to measure sulfate concentration using a calibrated sulfate photometer (HANNA HI93751). Sulfide, thiosulfate, and polysulfide concentrations were measured by argentimetric potentiometric titration [34] using an automatic titrator (848 Titrino Plus, Metrohm). Silver nitrate was used as the titrant. The titrator was equipped with a calibrated silver/silver sulfide ion selective electrode for sulfide determination and a calibrated iodide electrode with Ag/AgCl reference electrode for thiosulfate determination.

Measurement of pH, dissolved oxygen (DO), and temperature inside the bioreactor and the settler was carried out using Orion 4-Star meter (Thermo Scientific) equipped with a calibrated ROSS Ultra pH electrode and a calibrated polarographic dissolved oxygen probe. Measurement of pH and temperature outside the bioreactor was carried out with a calibrated Handylab 1 pH meter (Schott) and a Fisher Scientific digital thermometer, respectively.

H_2_S inlet and outlet gas concentrations were monitored by a H_2_S gas detector (BW GasAlertMax XT) with a measuring range of 0–200 ppm. Dilution of the gas in a dual-valve Tedlar PVF bag (Cole-Parmer EW-01409-93) with subsequent measurement was conducted when necessary.

## 3. Results and Discussion

### 3.1. Bioreactor Performance at Various H_**2**_S Loading Rates and pH

The H_2_S loading rate (LR), elimination capacity (EC), and removal efficiency (RE) of the bioreactor were calculated using the following equations:

(1) $$\begin{array}{c} \text{LR}=\frac{C_{gi}\times Q_{g}}{V_{R}},\\ \text{EC}=\frac{\left[\left(C_{gi}-C_{go}\right)\times Q_{g}-C_{lo}\times Q_{lo}\right]}{V_{R}},\\ \text{RE}=\frac{\left[\left(C_{gi}-C_{go}\right)\times 100\right]}{C_{gi}}, \end{array}$$

where *C*_*gi*_ and *C*_*go*_ are the inlet and outlet gaseous sulfide concentrations (g/m^3^), *C*_*lo*_ is the liquid discharge sulfide concentration (g/m^3^), *Q*_*g*_ is the volumetric gas flow rate (m^3^/h), *Q*_*lo*_ is the volumetric bioreactor liquid discharge flow rate (m^3^/h), and *V*_*R*_ is the working volume of the bioreactor (m^3^).

**Figure 2:**
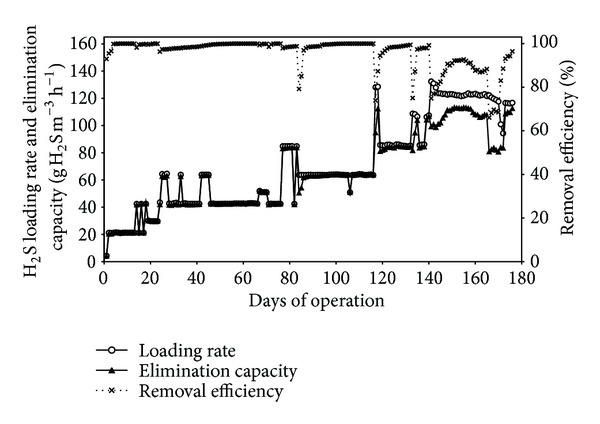
Daily performance of the airlift bioreactor over the study period.

Figure 2 shows H_2_S loading rates and elimination capacities as well as the removal efficiency during a 176-day period of continuous operation. Hydrogen sulfide loading rate was increased gradually up to 132.4 g H_2_S m^−3^ h^−1^ at day 141 and then decreased down to about 116 g H_2_S m^−3^ h^−1^ during the remaining period. Elimination capacities up to about 113 g H_2_S m^−3^ h^−1^ were attained during the study period. In terms of H_2_S gas removal efficiency, higher than 99% could be achieved at loading rates up to 108 g H_2_S m^−3^ h^−1^.

**Figure 3:**
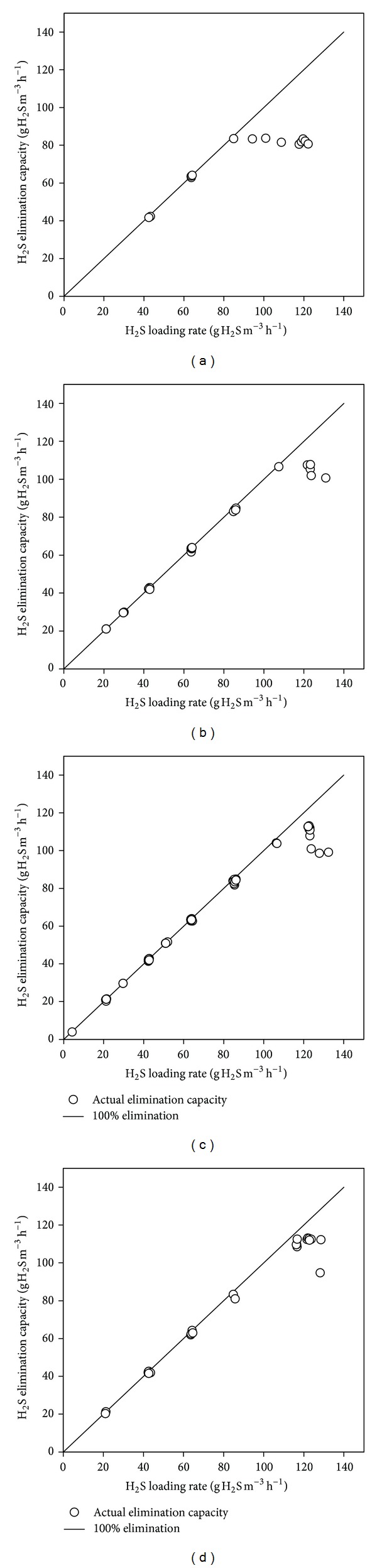
Effect of pH on the maximum elimination capacity of the airlift bioreactor: (a) pH = 6.5–6.9; (b) pH = 7.0–7.4; (c) pH = 7.5–7.9; and (d) pH = 8.0–8.5.

The effect of pH on the elimination capacity of the bioreactor at steady-state condition is illustrated in Figure 3. In all cases the elimination capacity of the bioreactor increased as the loading rate increased up to a maximum value beyond which a decrease in elimination capacity was observed. The maximum elimination capacities achieved at the studied pH ranges were about 84, 108, 113, and 113 g H_2_S m^−3^ h^−1^ at pH ranges 6.5–6.9, 7.0–7.4, 7.5–7.9, and 8.0–8.5, respectively. These maximum elimination capacities were achieved at loading rates in the range 120–130 g H_2_S m^−3^ h^−1^.

Fernández et al. [26] found similar trend with pH. However, their biotrickling filter was sensitive to H_2_S overloads at pH higher than 7.5, which was not the case with the current SOB. The close results at various pH ranges, particularly at pH >7, suggest that the SOB used in the bioreactor was capable of sustaining a wide pH range. This might be explained by the fact that the used SOB originated from a mixed culture rather than being a pure culture. The mixed culture contained several species of SOB allowing for adaptation to various environmental conditions.

The relatively low performance of the SOB at the lower pH range (below 7.0) might be attributed to biological capacity and/or mass-transfer limitation. H_2_S is an acidic gas that dissolves in alkaline solutions with a rate higher than that in acidic ones. Therefore, pH values in the alkaline range allow more H_2_S to dissolve and consequently be available for the existing SOB. On the other hand, low pH values might affect the SOB performance due to existence of higher concentrations of free or unionized sulfide in the solution as will be discussed later in Section 3.4.

The maximum elimination capacity achieved in the current airlift bioreactor was higher than other bioreactor configurations [2, 3, 5, 6] and comparable to others [25, 27] (Table 1). However, it was lower than other airlift bioreactors where sulfide solution rather than H_2_S gas was used as a feed [29, 35]. One reason for that may be the absence of mass-transfer problems in the liquid sulfide-fed airlift reactors compared to the gas-fed ones. This implies that application of H_2_S gas-fed airlift bioreactors might require reactor volumes larger than those in the sulfide-fed ones. However, gas-fed bioreactors eliminate the use of additional absorption column to convert H_2_S gas to sulfide solution and, thus, save the associated capital and operating costs. Also, the elimination capacity of the current bioreactor was lower than that of a biotrickling filter with polyurethane foam (PUF) packing [26] because of the higher mass-transfer rate provided by the large specific surface area of PUF. However, the disadvantage of this type of packing is pore clogging by the formed sulfur particles, which might raise maintenance problems.

### 3.2. The Effect of Oxygen Availability on Bioconversion End Product

**Figure 4:**
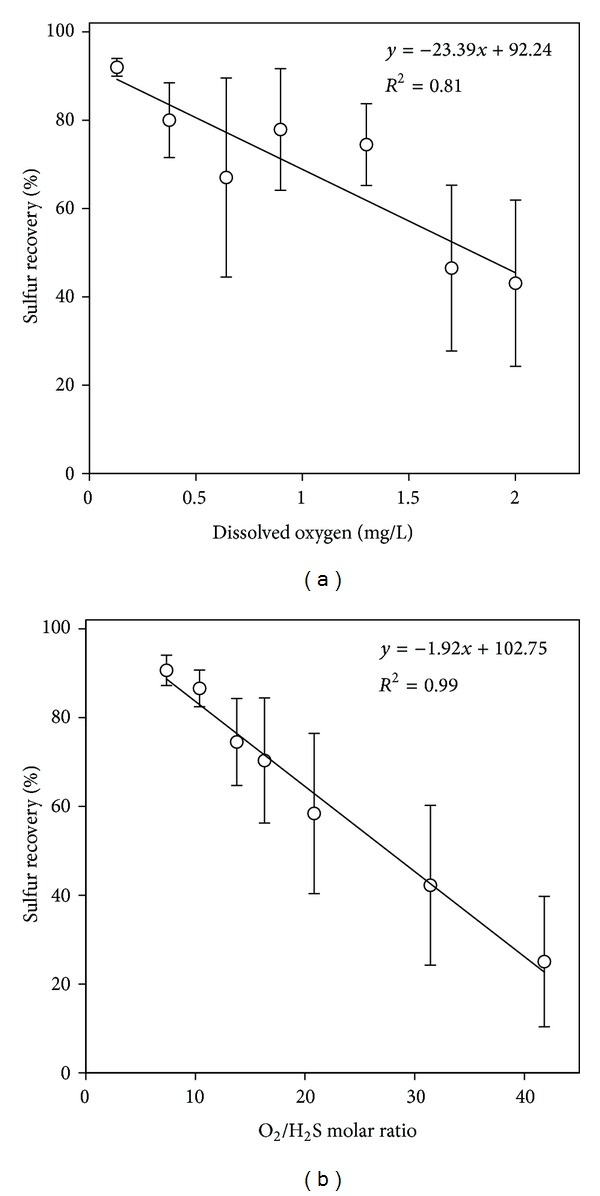
Effect of oxygen availability as (a) DO and (b) O_2_/H_2_S molar ratio on sulfur recovery.

The effect of oxygen availability as DO in the bioreactor solution is presented in Figure 4(a) which shows that elemental sulfur is the dominant end product at low DO. For instance, higher than 90% sulfur recovery (i.e., conversion of H_2_S into elemental sulfur) could be achieved at DO lower than 0.3 mg/L. As the DO concentration was increased sulfate formation increased on the expense of sulfur recovery. Sulfur recovery was lower than 40% at DO concentrations higher than 2 mg/L. Similar results were found by Lohwacharin and Annachhatre [29].

Buisman et al. [28] reported that biological oxidation of sulfide to sulfate proceeds in two stages as follows:

(2) $$\begin{array}{c} {\text{HS}}^{-}\overset{\left[\text{O}\right]+\text{SOB}}{⟶}\text{membrane}\;\text{bound}\;\left[{\text{S}}^{\text{o}}\right]⟷{\text{S}}^{\text{o}} \end{array}$$

(3) $$\begin{array}{c} \text{membrane}\;\text{bound}\left[{\text{S}}^{\text{o}}\right]\overset{\left[3\text{O}\right]}{⟶}{{\text{SO}}_{3}}^{-2}\overset{\left[\text{O}\right]}{⟶}{{\text{SO}}_{4}}^{-2} \end{array}$$

In the first stage, which proceeds faster than the second stage, sulfide looses two electrons and membrane-bound polymeric sulfur compounds are being formed (2). In the second step, this sulfur is oxidized to sulfite and then to sulfate (3). The higher oxidized forms are formed only if the amount of available oxygen is sufficient. If oxygen extent is controlled for achieving the first stage only, elemental sulfur will be the end product of the process.

Sulfate is not preferred as end product because of its adverse effect on sewerage system and may constitute a secondary pollutant. On the other hand, elemental sulfur (S^o^) is a noncorrosive solid that is easy to handle and transport. In addition, it has a commercial value exceeding that of sulfuric acid (or sulfate) [36]. Therefore, direction of bioconversion of H_2_S towards elemental sulfur formation is preferred.

It was reported in many published work that bioconversion of the inlet sulfide can be limited to elemental sulfur by maintaining DO concentration at <0.1 mg/L [37–39]. The performance of aerobic SOB as related to the available DO might be common to all bioreactor systems. However, bioreactors might differ from each other in the operational conditions to attain such low DO concentrations. It might be easy to control oxygen limited condition in an airlift bioreactor fed with liquid sulfide solutions by controlling the air dose to the bioreactor medium. On the other hand, in an airlift bioreactor fed with H_2_S-air mixture the DO concentration depends on many factors, of which O_2_/H_2_S molar ratio in the feed gas stream and mass-transfer are important. Therefore, it was important to study the relationship between O_2_/H_2_S molar ratio and the bioreactor performance in terms of % sulfur recovery and DO, which is specific for each airlift bioreactor.

The effect of O_2_/H_2_S molar ratio on % sulfur recovery is shown in Figure 4(b). Sulfur recovery increased at lower O_2_/H_2_S molar ratios. Higher than 90% conversion to elemental sulfur was achieved at O_2_/H_2_S molar ratios lower than 10. On the other hand, sulfate was the dominant end product at O_2_/H_2_S molar ratios > 20.

Compared to other bioreactors, the O_2_/H_2_S molar ratio that achieved maximum sulfur recovery in this study was found to be higher. In two of the other bioreactors [1, 28] packing material (e.g., polyurethane foam and polypropylene grid) was used to enhance mass-transfer of both H_2_S and oxygen. However, these types of packing materials may suffer from clogging by sulfur particles. In another bioreactor [30] sulfide solution and air were mixed in a separate stirred vessel, which might add to the operating cost of the bioreactor.

**Figure 5:**
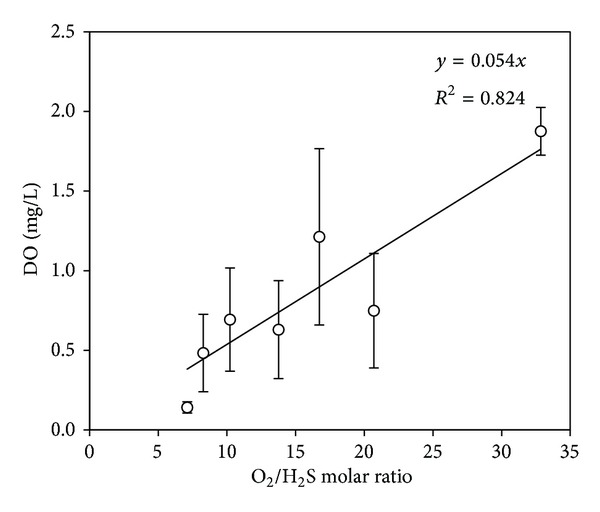
Correlation between O_2_/H_2_S molar ratio and DO.

This comparison indicates that oxygen availability in the cell suspension is a function of mass-transfer.  Figure 5 shows the relationship between the inlet O_2_/H_2_S molar ratio and dissolved oxygen, which is a characteristic of the current airlift bioreactor. An improvement in mass-transfer is expected to increase the slope of the linear equation.

The maximum conversion of H_2_S into elemental sulfur achieved in the airlift bioreactor with the current configuration was 95%, which is comparable to that achieved in some studies [1, 40] while being much higher than in others [29, 41].

During the last three months of the bioreactor operation the average percentage of H_2_S converted into thiosulfate was 0.67 ± 0.11%, mainly due to auto-oxidation of sulfide [30, 41] and/or reaction of sulfur with OH^−^ ion in alkaline solution [34, 42, 43]. The highest conversion to thiosulfate was obtained at higher O_2_/H_2_S molar ratios. Additionally, an average of 2.1% of the inlet sulfide was detected as sulfide in the outlet solution, which is very close to that reported by Fortuny et al. [1].

### 3.3. The Effect of pH on Bioconversion End Product

**Figure 6:**
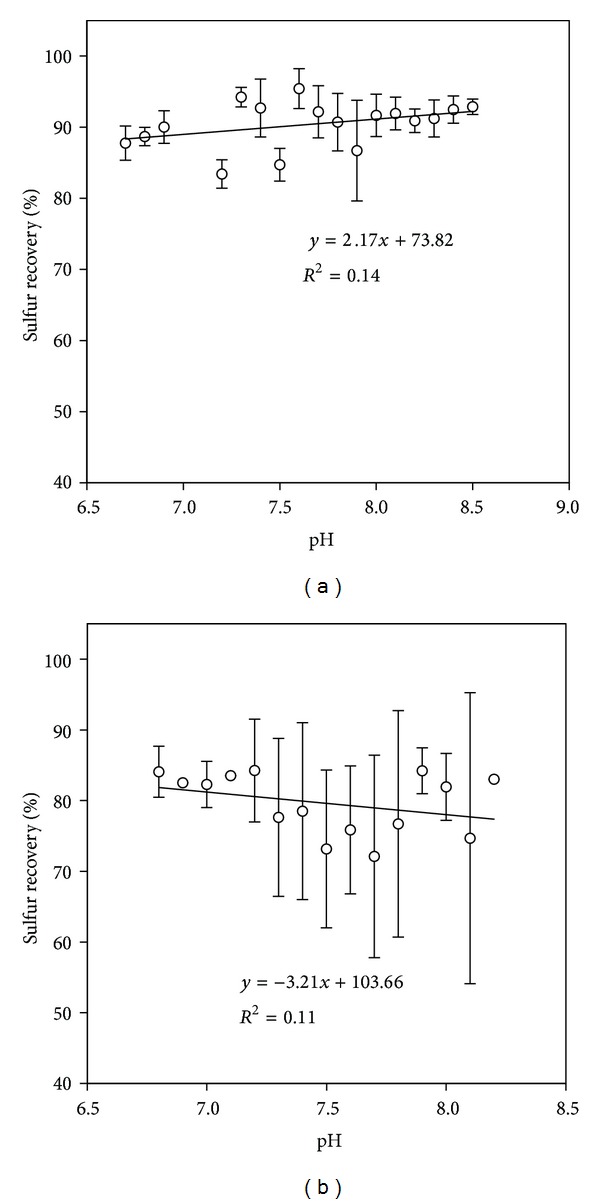
Effect of pH on bioconversion end product: (a) O_2_/H_2_S molar ratio <10 and (b) O_2_/H_2_S molar ratio 10–20.

The effect of pH on sulfur recovery was observed under oxygen-limited conditions (Figure 6(a)) and under excess oxygen (Figure 6(b)). At oxygen-limited conditions there was a slight increase of % sulfur recovery as the pH was increased. On the other hand, a decreased % sulfur recovery was observed at higher pH when oxygen was in excess.

It was found in previous studies that sulfur reacts with OH^−^ ion in alkaline solution according to the following equation [34]:

(4) $$\begin{array}{c} \left(4+2x\right)\text{S}+{6\text{OH}}^{-}⟶2{{\text{S}}_{x+1}}^{2-}+{\text{S}}_{2}{{\text{O}}_{3}}^{2-}+3{\text{H}}_{2}\text{O}. \end{array}$$

In the presence of excess sulfur (i.e., *x* > 0), which is the case at oxygen-limited conditions, polysulfide forms [42]. In this study polysulfide was included in elemental sulfur concentration since S^o^ concentration was calculated by mass balance, taking into account the inlet sulfide and the outlet sulfide, sulfate, and thiosulfate. This might explain the increasing trend of sulfur recovery with pH at oxygen-limited conditions (Figure 6(a)). On the other hand, at excessive oxygen conditions the produced elemental sulfur in the bioreactor was less. According to (4), less sulfur might result in sulfide formation on the expense of elemental sulfur. This might explain the decrease of sulfur recovery at high pH and excess oxygen (Figure 6(b)).

### 3.4. Effect of Accumulated Sulfide Concentration on Bioconversion Efficiency

**Figure 7:**
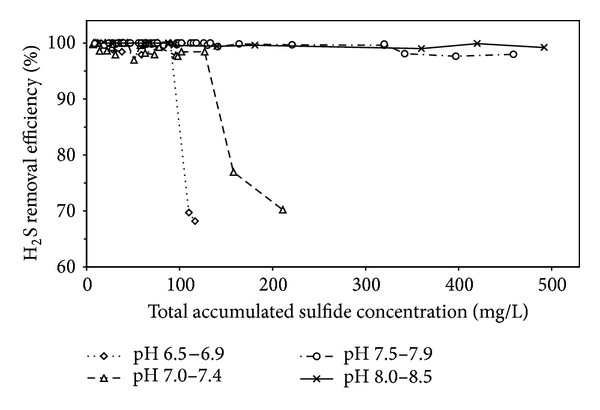
Effect of accumulated sulfide on bioconversion efficiency at various pH ranges.

The performance of the bioreactor in terms of H_2_S removal efficiency at four pH ranges and various accumulated sulfide concentrations is illustrated in Figure 7. The removal efficiency sharply dropped below 90% when the total accumulated sulfide concentration exceeded about 100 and 150 mg/L at pH ranges 6.5–6.9 and 7.0–7.4, respectively. The bioreactor performance severely dropped at higher accumulated sulfide concentrations. On the other hand, much higher concentrations of accumulated sulfide were tolerated at higher pH ranges. For instance, the removal efficiency was slightly affected under accumulated sulfide concentrations higher than 320 mg/L at pH range 7.5–7.9, however, remaining higher than 97%. At pH range of 8.0–8.5 the removal efficiency was not affected even at accumulated sulfide concentrations up to about 500 mg/L. Higher concentrations were not studied.

The combined effect of both accumulated sulfide and pH might be explained by three factors: (a) mass-transfer, (b) biological activity, and (c) the presence of unionized sulfide. H_2_S is an acidic gas that is expected to be absorbed in the bioreactor solution more easily at high pH values. Unless the resident SOB is capable of consuming the absorbed H_2_S gas dissolved sulfide will accumulate up to levels that are harmful to the resident microorganisms. Sulfide is toxic at higher concentrations for many bacteria. The inhibitory effect of sulfides presumed to be caused by unionized H_2_S because only neutral molecules can permeate well through the cell membrane [44].

The fraction of unionized H_2_S of the total sulfide is very much dependent on pH. Hydrogen sulfide is a diprotic acid that dissociate in two steps:

(5) H2S⟷H++HS−K1=[H+][HS−][H2S]=10−7 Mol L−1 at  20C°

(6) HS−⟷H++S−2K2=[H+][S−2][HS−]=0.8×10−17 Mol L−1 at  20C°.

Since the dissociation constant *K*_2_ is always so low (other values are reported), the equilibrium with S^−2^ can be neglected at intermediate pH values [45]. Therefore, at neutral to slightly alkaline conditions, only the equilibrium between H_2_S and HS^−^ is considered.

*K*_1_ is the dissociation constant. Its value changes with temperature (*T* °K) according to [33]:

(7) $$\begin{array}{c} pK_{1}=32.55+\frac{1519.44}{T}-15.672\;\log_{10}\;T+0.02722T,\\ pK_{1}=-\log_{10}\;K_{1}. \end{array}$$

The unionized H_2_S fraction of the total dissolved sulfide (*f*) can be calculated using *K*_1_ and pH values according to the following equation [46]:

(8) $$\begin{array}{c} f={\left(1+\frac{K_{1}}{10^{-\text{pH}}}\right)}^{-1}. \end{array}$$

The accumulated sulfide concentrations 100 mg/L (at pH range 6.5–6.9, average 6.7) and 150 mg/L (at pH range 7.0–7.4, average 7.2) beyond which inhibition of the SOB started (Figure 7) correspond to unionized H_2_S fractions of 0.63 and 0.35, respectively. These are equivalent to unionized sulfide concentrations of 63 and 52.5 mg/L, respectively. The 50% inhibitive unionized sulfide concentration was not studied but is expected to be higher than these two concentrations. Considering the least unionized sulfide concentration (52.5 mg/L), the equivalent total sulfide that can be tolerated by the SOB at pH ranges 7.5–7.9 (average: 7.7) and 8.0–8.5 (average 8.2) is expected to be 375 and 1050 mg/L, respectively.

Using Henry's law at 30°C (partition coefficient is about 2.0), the gas phase concentration of H_2_S that can be tolerated without inhibition of the resident SOB can be calculated as about 38000, 57000, 142000, and 396000 ppm at pH values of 6.7, 7.2, 7.7, and 8.2, respectively, assuming optimum mass-transfer rate and the presence of sufficient microorganisms to consume the absorbed H_2_S.

## 4. Conclusion

A maximum H_2_S elimination capacity of 113 g H_2_S m^−3^ h^−1^ was achieved in the airlift bioreactor under investigation at loading rates up to 130 g H_2_S m^−3^ h^−1^, a result indicating the feasibility of using such bioreactor in biotreatment of high concentrations of H_2_S in air streams directly injected into the bioreactor.

pH is an important parameter that should be adjusted for better performance of the bioreactor. The effect of pH in association with other factors on the bioreactor performance was studied. It was found that the current airlift bioreactor (with the resident SOB) was capable of achieving almost the same H_2_S elimination capacity at a wide range of pH, particularly 7–8.5. At lower pH values, the elimination capacity was lower.

The bioreactor achieved maximum elemental sulfur recovery (about 95%) under oxygen limited conditions (DO below 0.2 mg/L). At low DO levels, higher pH values increased elemental sulfur recovery.

The resident SOB in the bioreactor tolerated accumulated sulfide concentrations higher than 500 mg/L at higher pH values (8.0–8.5) and near 100% removal efficiency was achieved. However, lower pH reduced the maximum tolerated accumulated sulfide in cell suspension.

The overall conclusion is, therefore, that the resident SOB in the studied airlift bioreactor favored pH values in the slightly alkaline range. The bioreactor performance in terms of elimination capacity and sulfur recovery was better at the alkaline pH range 8–8.5. The ability of the airlift bioreactor used in this study to handle the high inlet concentrations of H_2_S is a proof that it can be a promising option for treatment of gas streams such as biogas from anaerobic digesters or landfills which may contain H_2_S concentrations up to several thousand ppm. However, more studies are recommended to apply gas streams with composition similar to that emitted from such processes.

## Figures and Tables

**Table 1:** Comparison between the maximum elimination capacity of the current airlift bioreactor and other studies.

Type of bioreactor	Sulfide feed form	Maximum EC	Reference
Biofilter packed with sodium alginate beads	H_2_S gas	8 g H_2_S m^−3^ h^−1^	[3]
Fixed film bioscrubber	H_2_S gas	19.4 g H_2_S m^−3^ h^−1^	[5]
Biofilter packed with organic materials	H_2_S gas	79 g H_2_S m^−3^ h^−1^	[6]
Biotrickling filter packed with polyurethane foam	H_2_S gas	55 g S m^−3^ h^−1^	[2]
Biofilter packed with GAC	H_2_S gas	125 g H_2_S m^−3^ h^−1^	[25]
Biotrickling filter packed with polyurethane foam	H_2_S gas	170 g S m^−3^ h^−1^	[26]
Industrial scale biotrickling filter packed with polypropylene Pall rings	H_2_S gas	110 g H_2_S m^−3^ h^−1^	[27]
Airlift bioreactor	Sulfide solution	4.3 kg S/kg VSS*·*d (≈160 g S m^−3^ h^−1^)	[29]
Airlift bioreactor	Sulfide solution	6.7 mol/m^3^ *·*h (214.4 g S m^−3^ h^−1^)	[35]
Airlift bioreactor	H_2_S gas	113 g H_2_S m^−3^ h^−1^	This study
